# The Inventory of Psychotic-Like Anomalous Self-Experiences (IPASE): an easy tool for investigating Self-Disorders, subjective experiences and global functioning

**DOI:** 10.1192/j.eurpsy.2023.598

**Published:** 2023-07-19

**Authors:** F. Magnani, S. Amorosi, C. Dell’Anna, V. Lucarini, M. Ballerini, C. Marchesi, M. Tonna

**Affiliations:** ^1^Department of Medicine and Surgery, Unit of Neuroscience, Parma University Hospital, Parma, Italy; ^2^Institute of Psychiatry and Neuroscience of Paris (IPNP), Université Paris Cité, Paris, France; ^3^Department of Mental Health, Local Health Service, Firenze; ^4^Department of Mental Health, Local Health Service, Parma, Italy

## Abstract

**Introduction:**

Self Disorders (SDs) are regarded as the subjective phenotype of Schizophrenia vulnerability. The EASE (Examination of Anomalous Self-Experiences) scale is the most detailed and widely used instrument to investigate SDs, but it requires long administration times and specific training. The IPASE (Inventory of Psychotic-like Anomalous Self-Experiences) scale might be a self-administered instrument of widespread use for an easier SDs investigation.

**Objectives:**

The present study was aimed at validating the Italian version of IPASE, testing its internal consistency and usability for a first level SDs survey. A secondary objective was to confirm the correlations between IPASE, EASE, main symptom dimensions, subjective bodily experiences, symptoms of schizophrenic autism as well as levels of global functioning.

**Methods:**

Fifty patients with Schizophrenia were administered the IPASE scale in its Italian version, the Examination of Anomalous Self-Experiences scale (EASE), the Positive And Negative Symptoms Scale (PANSS), the Social and Occupational Functioning Assessment Scale (SOFAS) to assess global functioning, the Autism Rating Scale (ARS) and the Abnormal Bodily Phenomena questionnaire (ABPq). The internal consistency of IPASE in its Italian version was investigated and the correlations between IPASE, EASE, ABP, ARS, PANSS and SOFAS were explored.

**Results:**

The internal consistency of the Italian version of IPASE was high (α 0.97). The IPASE and EASE total scores were positively correlated with each other, as were many of the conceptually related subdomains of both scales. The IPASE score was negatively correlated with global functioning (SOFAS) and positively correlated with total PANSS scores and with PANSS negative domain. Moreover, the IPASE total score was positively correlated with autism dimension (ARS), while anomalies in subjective experience of the lived body were coherently correlated with higher scores in IPASE “somatization” subdomain.

**Conclusions:**

The IPASE may be an easy instrument with high internal consistency for an initial investigation of SDs. IPASE domains appear to be correlated with the SDs investigated through EASE and with the main symptomatologic dimensions of Schizophrenia, in particular with negative symptoms. IPASE might also be a useful instrument for a first level investigation of subjective experiences concerning intersubjectivity and bodily dimensions.

SDs are confirmed to be a core feature of the schizophrenia psychopathology, with a adverse impact on global functioning.

**Disclosure of Interest:**

None Declared

